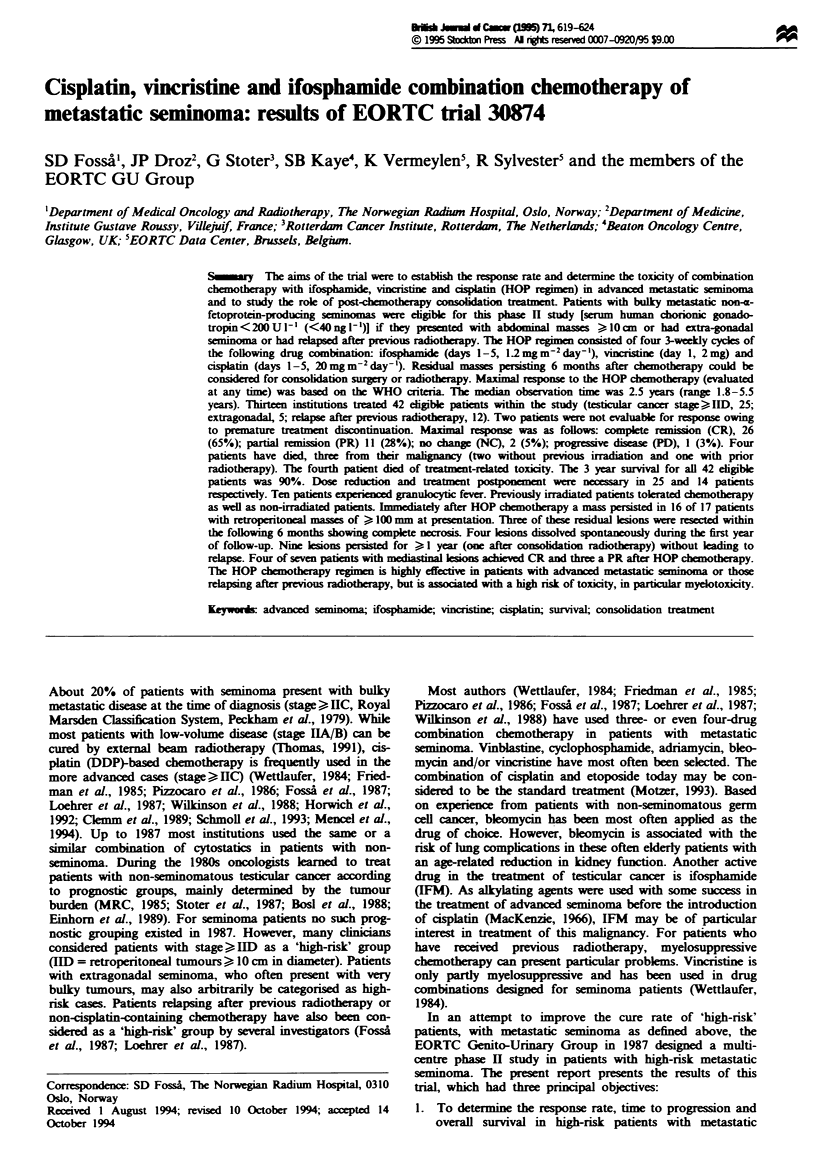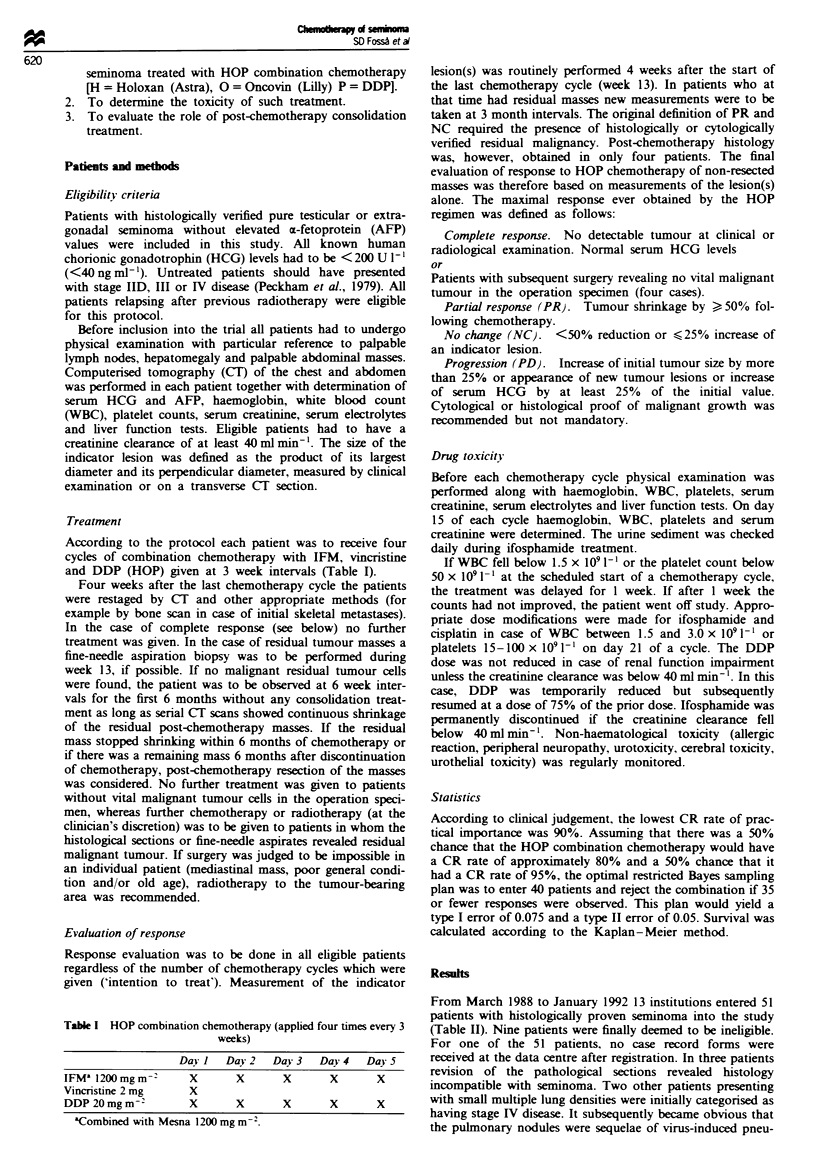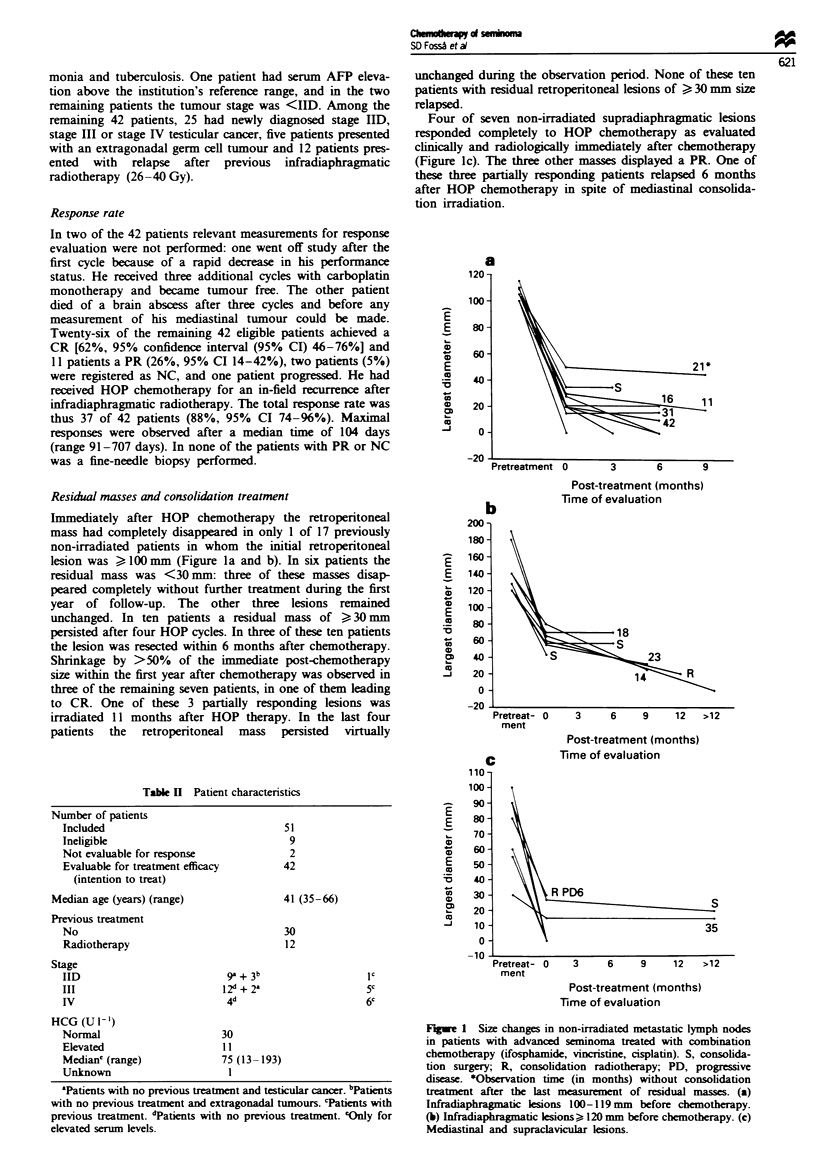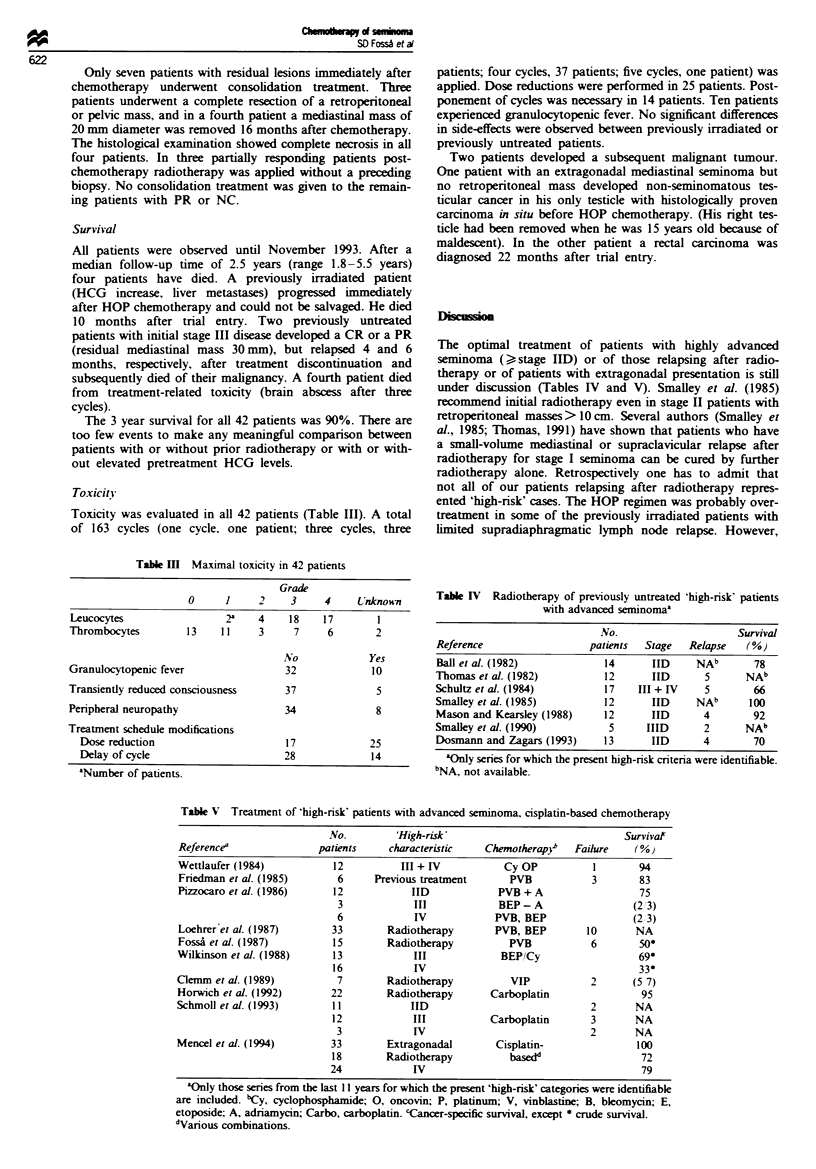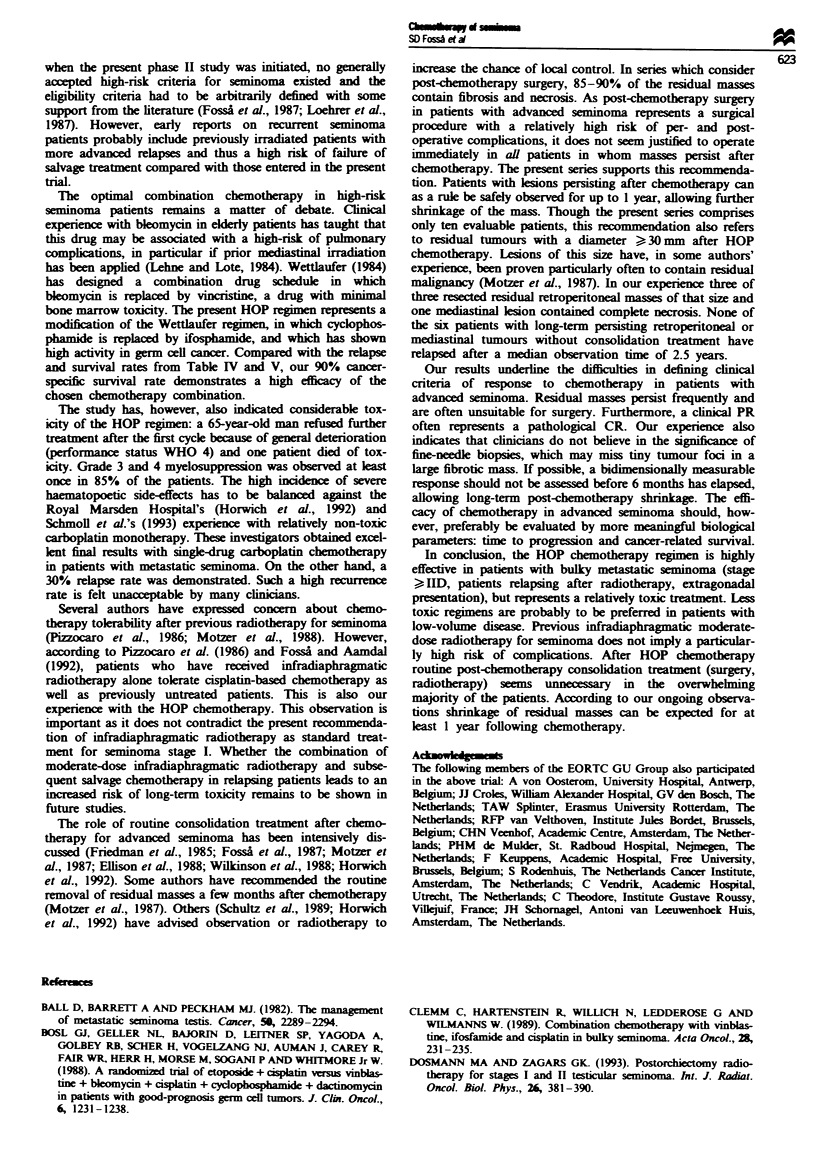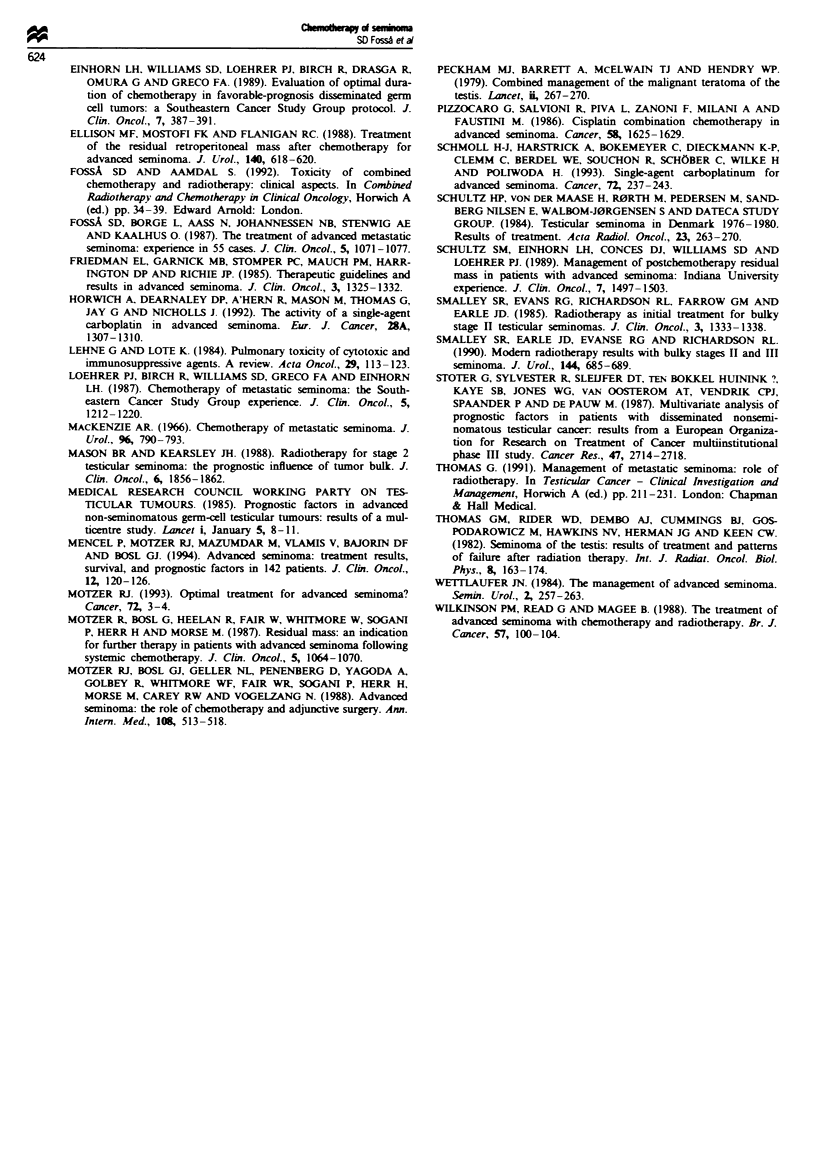# Cisplatin, vincristine and ifosphamide combination chemotherapy of metastatic seminoma: results of EORTC trial 30874. EORTC GU Group.

**DOI:** 10.1038/bjc.1995.121

**Published:** 1995-03

**Authors:** S. D. Fosså, J. P. Droz, G. Stoter, S. B. Kaye, K. Vermeylen, R. Sylvester

**Affiliations:** Department of Medical Oncology and Radiotherapy, Norwegian Radium Hospital, Oslo.

## Abstract

The aims of the trial were to establish the response rate and determine the toxicity of combination chemotherapy with ifosphamide, vincristine and cisplatin (HOP regimen) in advanced metastatic seminoma and to study the role of post-chemotherapy consolidation treatment. Patients with bulky metastatic non-alpha-fetoprotein-producing seminomas were eligible for this phase II study [serum human chorionic gonadotropin < 200 U l-1 (< 40 ng l-1)] if they presented with abdominal masses > or = 10 cm or had extra-gonadal seminoma or had relapsed after previous radiotherapy. The HOP regimen consisted of four 3-weekly cycles of the following drug combination: ifosphamide (days 1-5, 1.2 mg m-2 day-1), vincristine (day 1, 2 mg) and cisplatin (days 1-5, 20 mg m-2 day-1). Residual masses persisting 6 months after chemotherapy could be considered for consolidation surgery or radiotherapy. Maximal response to the HOP chemotherapy (evaluated at any time) was based on the WHO criteria. The median observation time was 2.5 years (range 1.8-5.5 years). Thirteen institutions treated 42 eligible patients within the study (testicular cancer stage > or = IID, 25; extragonadal, 5; relapse after previous radiotherapy, 12). Two patients were not evaluable for response owing to premature treatment discontinuation. Maximal response was as follows: complete remission (CR), 26 (65%); partial remission (PR) 11 (28%); no change (NC), 2 (5%); progressive disease (PD), 1 (3%). Four patients have died, three from their malignancy (two without previous irradiation and one with prior radiotherapy). The fourth patient died of treatment-related toxicity. The 3 year survival for all 42 eligible patients was 90%. Dose reduction and treatment postponement were necessary in 25 and 14 patients respectively. Ten patients experienced granulocytic fever. Previously irradiated patients tolerated chemotherapy as well as non-irradiated patients. Immediately after HOP chemotherapy a mass persisted in 16 of 17 patients with retroperitoneal masses of > or = 100 mm at presentation. Three of these residual lesions were resected within the following 6 months showing complete necrosis. Four lesions dissolved spontaneously during the first year of follow-up. Nine lesions persisted for > or = 1 year (one after consolidation radiotherapy) without leading to relapse. Four of seven patients with mediastinal lesions achieved CR and three a PR after HOP chemotherapy. The HOP chemotherapy regimen is highly effective in patients with advanced metastatic seminoma or those relapsing after previous radiotherapy, but is associated with a high risk of toxicity, in particular myelotoxicity.


© 1995 Stockton Press All rights reserved 0007–0920/95 $9.00

### Cisplatin, vincristine and ifosphamide combination chemotherapy of metastatic seminoma: results of EORTC trial 30874

SD Fosså[1], JP Droz[2], G Stoter[3], SB Kaye[4], K Vermeylen[5], R Sylvester[5] and the members of the EORTC GU Group

[1]*Department of Medical Oncology and Radiotherapy, The Norwegian Radium Hospital, Oslo, Norway;* [2]*Department of Medicine, Institute Gustave Roussy, Villejuif, France;* [3]*Rotterdam Cancer Institute, Rotterdam, The Netherlands;* [4]*Beaton Oncology Centre, Glasgow, UK;* [5]*EORTC Data Center, Brussels, Belgium.*

**Summary** The aims of the trial were to establish the response rate and determine the toxicity of combination chemotherapy with ifosphamide, vincristine and cisplatin (HOP regimen) in advanced metastatic seminoma and to study the role of post-chemotherapy consolidation treatment. Patients with bulky metastatic non-α-fetoprotein-producing seminomas were eligible for this phase II study [serum human chorionic gonadotropin < 200 U $l^{-1}$ (< 40 ng $l^{-1}$)] if they presented with abdominal masses ⩾ 10 cm or had extra-gonadal seminoma or had relapsed after previous radiotherapy. The HOP regimen consisted of four 3-weekly cycles of the following drug combination: ifosphamide (days 1–5, 1.2 mg $m^{-2}$ $day^{-1}$), vincristine (day 1, 2 mg) and cisplatin (days 1–5, 20 mg $m^{-2}$ $day^{-1}$). Residual masses persisting 6 months after chemotherapy could be considered for consolidation surgery or radiotherapy. Maximal response to the HOP chemotherapy (evaluated at any time) was based on the WHO criteria. The median observation time was 2.5 years (range 1.8–5.5 years). Thirteen institutions treated 42 eligible patients within the study (testicular cancer stage ⩾ IID, 25; extragonadal, 5; relapse after previous radiotherapy, 12). Two patients were not evaluable for response owing to premature treatment discontinuation. Maximal response was as follows: complete remission (CR), 26 (65%); partial remission (PR) 11 (28%); no change (NC), 2 (5%); progressive disease (PD), 1 (3%). Four patients have died, three from their malignancy (two without previous irradiation and one with prior radiotherapy). The fourth patient died of treatment-related toxicity. The 3 year survival for all 42 eligible patients was 90%. Dose reduction and treatment postponement were necessary in 25 and 14 patients respectively. Ten patients experienced granulocytic fever. Previously irradiated patients tolerated chemotherapy as well as non-irradiated patients. Immediately after HOP chemotherapy a mass persisted in 16 of 17 patients with retroperitoneal masses of ⩾ 100 mm at presentation. Three of these residual lesions were resected within the following 6 months showing complete necrosis. Four lesions dissolved spontaneously during the first year of follow-up. Nine lesions persisted for ⩾ 1 year (one after consolidation radiotherapy) without leading to relapse. Four of seven patients with mediastinal lesions achieved CR and three a PR after HOP chemotherapy. The HOP chemotherapy regimen is highly effective in patients with advanced metastatic seminoma or those relapsing after previous radiotherapy, but is associated with a high risk of toxicity, in particular myelotoxicity.

**Keywords:** advanced seminoma; ifosphamide; vincristine; cisplatin; survival; consolidation treatment

About 20% of patients with seminoma present with bulky metastatic disease at the time of diagnosis (stage ⩾ IIC, Royal Marsden Classification System, Peckham *et al.*, 1979). While most patients with low-volume disease (stage IIA/B) can be cured by external beam radiotherapy (Thomas, 1991), cisplatin (DDP)-based chemotherapy is frequently used in the more advanced cases (stage ⩾ IIC) (Wettlaufer, 1984; Friedman *et al.*, 1985; Pizzocaro *et al.*, 1986; Fosså *et al.*, 1987; Loehrer *et al.*, 1987; Wilkinson *et al.*, 1988; Horwich *et al.*, 1992; Clemm *et al.*, 1989; Schmoll *et al.*, 1993; Mencel *et al.*, 1994). Up to 1987 most institutions used the same or a similar combination of cytostatics in patients with non-seminoma. During the 1980s oncologists learned to treat patients with non-seminomatous testicular cancer according to prognostic groups, mainly determined by the tumour burden (MRC, 1985; Stoter *et al.*, 1987; Bosl *et al.*, 1988; Einhorn *et al.*, 1989). For seminoma patients no such prognostic grouping existed in 1987. However, many clinicians considered patients with stage ⩾ IID as a 'high-risk' group (IID = retroperitoneal tumours ⩾ 10 cm in diameter). Patients with extragonadal seminoma, who often present with very bulky tumours, may also arbitrarily be categorised as high-risk cases. Patients relapsing after previous radiotherapy or non-cisplatin-containing chemotherapy have also been considered as a 'high-risk' group by several investigators (Fosså *et al.*, 1987; Loehrer *et al.*, 1987).

Most authors (Wettlaufer, 1984; Friedman *et al.*, 1985; Pizzocaro *et al.*, 1986; Fosså *et al.*, 1987; Loehrer *et al.*, 1987; Wilkinson *et al.*, 1988) have used three- or even four-drug combination chemotherapy in patients with metastatic seminoma. Vinblastine, cyclophosphamide, adriamycin, bleomycin and/or vincristine have most often been selected. The combination of cisplatin and etoposide today may be considered to be the standard treatment (Motzer, 1993). Based on experience from patients with non-seminomatous germ cell cancer, bleomycin has been most often applied as the drug of choice. However, bleomycin is associated with the risk of lung complications in these often elderly patients with an age-related reduction in kidney function. Another active drug in the treatment of testicular cancer is ifosphamide (IFM). As alkylating agents were used with some success in the treatment of advanced seminoma before the introduction of cisplatin (MacKenzie, 1966), IFM may be of particular interest in treatment of this malignancy. For patients who have received previous radiotherapy, myelosuppressive chemotherapy can present particular problems. Vincristine is only partly myelosuppressive and has been used in drug combinations designed for seminoma patients (Wettlaufer, 1984).

In an attempt to improve the cure rate of 'high-risk' patients, with metastatic seminoma as defined above, the EORTC Genito-Urinary Group in 1987 designed a multicentre phase II study in patients with high-risk metastatic seminoma. The present report presents the results of this trial, which had three principal objectives:

1. To determine the response rate, time to progression and overall survival in high-risk patients with metastatic

Correspondence: SD Fosså, The Norwegian Radium Hospital, 0310 Oslo, Norway
Received 1 August 1994; revised 10 October 1994; accepted 14 October 1994

seminoma treated with HOP combination chemotherapy [H = Holoxan (Astra), O = Oncovin (Lilly) P = DDP].
2. To determine the toxicity of such treatment.
3. To evaluate the role of post-chemotherapy consolidation treatment.

### Patients and methods

*Eligibility criteria*

Patients with histologically verified pure testicular or extragonadal seminoma without elevated α-fetoprotein (AFP) values were included in this study. All known human chorionic gonadotrophin (HCG) levels had to be $<200\ U\ l^{-1}$ ($<40\ ng\ ml^{-1}$). Untreated patients should have presented with stage IID, III or IV disease (Peckham *et al.*, 1979). All patients relapsing after previous radiotherapy were eligible for this protocol.

Before inclusion into the trial all patients had to undergo physical examination with particular reference to palpable lymph nodes, hepatomegaly and palpable abdominal masses. Computerised tomography (CT) of the chest and abdomen was performed in each patient together with determination of serum HCG and AFP, haemoglobin, white blood count (WBC), platelet counts, serum creatinine, serum electrolytes and liver function tests. Eligible patients had to have a creatinine clearance of at least $40\ ml\ min^{-1}$. The size of the indicator lesion was defined as the product of its largest diameter and its perpendicular diameter, measured by clinical examination or on a transverse CT section.

*Treatment*

According to the protocol each patient was to receive four cycles of combination chemotherapy with IFM, vincristine and DDP (HOP) given at 3 week intervals (Table I).

Four weeks after the last chemotherapy cycle the patients were restaged by CT and other appropriate methods (for example by bone scan in case of initial skeletal metastases). In the case of complete response (see below) no further treatment was given. In the case of residual tumour masses a fine-needle aspiration biopsy was to be performed during week 13, if possible. If no malignant residual tumour cells were found, the patient was to be observed at 6 week intervals for the first 6 months without any consolidation treatment as long as serial CT scans showed continuous shrinkage of the residual post-chemotherapy masses. If the residual mass stopped shrinking within 6 months of chemotherapy or if there was a remaining mass 6 months after discontinuation of chemotherapy, post-chemotherapy resection of the masses was considered. No further treatment was given to patients without vital malignant tumour cells in the operation specimen, whereas further chemotherapy or radiotherapy (at the clinician's discretion) was to be given to patients in whom the histological sections or fine-needle aspirates revealed residual malignant tumour. If surgery was judged to be impossible in an individual patient (mediastinal mass, poor general condition and/or old age), radiotherapy to the tumour-bearing area was recommended.

*Evaluation of response*

Response evaluation was to be done in all eligible patients regardless of the number of chemotherapy cycles which were given ('intention to treat'). Measurement of the indicator lesion(s) was routinely performed 4 weeks after the start of the last chemotherapy cycle (week 13). In patients who at that time had residual masses new measurements were to be taken at 3 month intervals. The original definition of PR and NC required the presence of histologically or cytologically verified residual malignancy. Post-chemotherapy histology was, however, obtained in only four patients. The final evaluation of response to HOP chemotherapy of non-resected masses was therefore based on measurements of the lesion(s) alone. The maximal response ever obtained by the HOP regimen was defined as follows:

*Complete response.* No detectable tumour at clinical or radiological examination. Normal serum HCG levels
*or*
Patients with subsequent surgery revealing no vital malignant tumour in the operation specimen (four cases).

*Partial response (PR).* Tumour shrinkage by ⩾50% following chemotherapy.

*No change (NC).* <50% reduction or ⩽25% increase of an indicator lesion.

*Progression (PD).* Increase of initial tumour size by more than 25% or appearance of new tumour lesions or increase of serum HCG by at least 25% of the initial value. Cytological or histological proof of malignant growth was recommended but not mandatory.

*Drug toxicity*

Before each chemotherapy cycle physical examination was performed along with haemoglobin, WBC, platelets, serum creatinine, serum electrolytes and liver function tests. On day 15 of each cycle haemoglobin, WBC, platelets and serum creatinine were determined. The urine sediment was checked daily during ifosphamide treatment.

If WBC fell below $1.5 \times 10^9\ l^{-1}$ or the platelet count below $50 \times 10^9\ l^{-1}$ at the scheduled start of a chemotherapy cycle, the treatment was delayed for 1 week. If after 1 week the counts had not improved, the patient went off study. Appropriate dose modifications were made for ifosphamide and cisplatin in case of WBC between 1.5 and $3.0 \times 10^9\ l^{-1}$ or platelets $15-100 \times 10^9\ l^{-1}$ on day 21 of a cycle. The DDP dose was not reduced in case of renal function impairment unless the creatinine clearance was below $40\ ml\ min^{-1}$. In this case, DDP was temporarily reduced but subsequently resumed at a dose of 75% of the prior dose. Ifosphamide was permanently discontinued if the creatinine clearance fell below $40\ ml\ min^{-1}$. Non-haematological toxicity (allergic reaction, peripheral neuropathy, urotoxicity, cerebral toxicity, urothelial toxicity) was regularly monitored.

*Statistics*

According to clinical judgement, the lowest CR rate of practical importance was 90%. Assuming that there was a 50% chance that the HOP combination chemotherapy would have a CR rate of approximately 80% and a 50% chance that it had a CR rate of 95%, the optimal restricted Bayes sampling plan was to enter 40 patients and reject the combination if 35 or fewer responses were observed. This plan would yield a type I error of 0.075 and a type II error of 0.05. Survival was calculated according to the Kaplan–Meier method.

### Results

From March 1988 to January 1992 13 institutions entered 51 patients with histologically proven seminoma into the study (Table II). Nine patients were finally deemed to be ineligible. For one of the 51 patients, no case record forms were received at the data centre after registration. In three patients revision of the pathological sections revealed histology incompatible with seminoma. Two other patients presenting with small multiple lung densities were initially categorised as having stage IV disease. It subsequently became obvious that the pulmonary nodules were sequelae of virus-induced pneu-

**Table I** HOP combination chemotherapy (applied four times every 3 weeks)

| | *Day 1* | *Day 2* | *Day 3* | *Day 4* | *Day 5* |
|---|---|---|---|---|---|
| IFM[a] 1200 mg m$^{-2}$ | X | X | X | X | X |
| Vincristine 2 mg | X | | | | |
| DDP 20 mg m$^{-2}$ | X | X | X | X | X |

[a]Combined with Mesna 1200 mg m$^{-2}$.

monia and tuberculosis. One patient had serum AFP elevation above the institution's reference range, and in the two remaining patients the tumour stage was <IID. Among the remaining 42 patients, 25 had newly diagnosed stage IID, stage III or stage IV testicular cancer, five patients presented with an extragonadal germ cell tumour and 12 patients presented with relapse after previous infradiaphragmatic radiotherapy (26–40 Gy).

*Response rate*

In two of the 42 patients relevant measurements for response evaluation were not performed: one went off study after the first cycle because of a rapid decrease in his performance status. He received three additional cycles with carboplatin monotherapy and became tumour free. The other patient died of a brain abscess after three cycles and before any measurement of his mediastinal tumour could be made. Twenty-six of the remaining 42 eligible patients achieved a CR [62%, 95% confidence interval (95% CI) 46–76%] and 11 patients a PR (26%, 95% CI 14–42%), two patients (5%) were registered as NC, and one patient progressed. He had received HOP chemotherapy for an in-field recurrence after infradiaphragmatic radiotherapy. The total response rate was thus 37 of 42 patients (88%, 95% CI 74–96%). Maximal responses were observed after a median time of 104 days (range 91–707 days). In none of the patients with PR or NC was a fine-needle biopsy performed.

*Residual masses and consolidation treatment*

Immediately after HOP chemotherapy the retroperitoneal mass had completely disappeared in only 1 of 17 previously non-irradiated patients in whom the initial retroperitoneal lesion was ⩾100 mm (Figure 1a and b). In six patients the residual mass was <30 mm: three of these masses disappeared completely without further treatment during the first year of follow-up. The other three lesions remained unchanged. In ten patients a residual mass of ⩾30 mm persisted after four HOP cycles. In three of these ten patients the lesion was resected within 6 months after chemotherapy. Shrinkage by >50% of the immediate post-chemotherapy size within the first year after chemotherapy was observed in three of the remaining seven patients, in one of them leading to CR. One of these 3 partially responding lesions was irradiated 11 months after HOP therapy. In the last four patients the retroperitoneal mass persisted virtually unchanged during the observation period. None of these ten patients with residual retroperitoneal lesions of ⩾30 mm size relapsed.

Four of seven non-irradiated supradiaphragmatic lesions responded completely to HOP chemotherapy as evaluated clinically and radiologically immediately after chemotherapy (Figure 1c). The three other masses displayed a PR. One of these three partially responding patients relapsed 6 months after HOP chemotherapy in spite of mediastinal consolidation irradiation.

**Table II** Patient characteristics

| | | |
|---|---|---|
| Number of patients | | |
| Included | 51 | |
| Ineligible | 9 | |
| Not evaluable for response | 2 | |
| Evaluable for treatment efficacy (intention to treat) | 42 | |
| Median age (years) (range) | 41 (35–66) | |
| Previous treatment | | |
| No | 30 | |
| Radiotherapy | 12 | |
| Stage | | |
| IID | 9[a] + 3[b] | 1[c] |
| III | 12[d] + 2[a] | 5[c] |
| IV | 4[d] | 6[c] |
| HCG ($U l^{-1}$) | | |
| Normal | 30 | |
| Elevated | 11 | |
| Median[e] (range) | 75 (13–193) | |
| Unknown | 1 | |

[a]Patients with no previous treatment and testicular cancer. [b]Patients with no previous treatment and extragonadal tumours. [c]Patients with previous treatment. [d]Patients with no previous treatment. [e]Only for elevated serum levels.

**Figure 1** Size changes in non-irradiated metastatic lymph nodes in patients with advanced seminoma treated with combination chemotherapy (ifosphamide, vincristine, cisplatin). S, consolidation surgery; R, consolidation radiotherapy; PD, progressive disease. *Observation time (in months) without consolidation treatment after the last measurement of residual masses. (**a**) Infradiaphragmatic lesions 100–119 mm before chemotherapy. (**b**) Infradiaphragmatic lesions ⩾120 mm before chemotherapy. (**c**) Mediastinal and supraclavicular lesions.

Only seven patients with residual lesions immediately after chemotherapy underwent consolidation treatment. Three patients underwent a complete resection of a retroperitoneal or pelvic mass, and in a fourth patient a mediastinal mass of 20 mm diameter was removed 16 months after chemotherapy. The histological examination showed complete necrosis in all four patients. In three partially responding patients post-chemotherapy radiotherapy was applied without a preceding biopsy. No consolidation treatment was given to the remaining patients with PR or NC.

*Survival*

All patients were observed until November 1993. After a median follow-up time of 2.5 years (range 1.8–5.5 years) four patients have died. A previously irradiated patient (HCG increase, liver metastases) progressed immediately after HOP chemotherapy and could not be salvaged. He died 10 months after trial entry. Two previously untreated patients with initial stage III disease developed a CR or a PR (residual mediastinal mass 30 mm), but relapsed 4 and 6 months, respectively, after treatment discontinuation and subsequently died of their malignancy. A fourth patient died from treatment-related toxicity (brain abscess after three cycles).

The 3 year survival for all 42 patients was 90%. There are too few events to make any meaningful comparison between patients with or without prior radiotherapy or with or without elevated pretreatment HCG levels.

*Toxicity*

Toxicity was evaluated in all 42 patients (Table III). A total of 163 cycles (one cycle, one patient; three cycles, three patients; four cycles, 37 patients; five cycles, one patient) was applied. Dose reductions were performed in 25 patients. Postponement of cycles was necessary in 14 patients. Ten patients experienced granulocytopenic fever. No significant differences in side-effects were observed between previously irradiated or previously untreated patients.

Two patients developed a subsequent malignant tumour. One patient with an extragonadal mediastinal seminoma but no retroperitoneal mass developed non-seminomatous testicular cancer in his only testicle with histologically proven carcinoma *in situ* before HOP chemotherapy. (His right testicle had been removed when he was 15 years old because of maldescent). In the other patient a rectal carcinoma was diagnosed 22 months after trial entry.

### Discussion

The optimal treatment of patients with highly advanced seminoma (≥stage IID) or of those relapsing after radiotherapy or of patients with extragonadal presentation is still under discussion (Tables IV and V). Smalley *et al.* (1985) recommend initial radiotherapy even in stage II patients with retroperitoneal masses > 10 cm. Several authors (Smalley *et al.*, 1985; Thomas, 1991) have shown that patients who have a small-volume mediastinal or supraclavicular relapse after radiotherapy for stage I seminoma can be cured by further radiotherapy alone. Retrospectively one has to admit that not all of our patients relapsing after radiotherapy represented 'high-risk' cases. The HOP regimen was probably overtreatment in some of the previously irradiated patients with limited supradiaphragmatic lymph node relapse. However,

**Table III** Maximal toxicity in 42 patients

| | Grade 0 | 1 | 2 | 3 | 4 | Unknown |
|---|---|---|---|---|---|---|
| Leucocytes | | 2[a] | 4 | 18 | 17 | 1 |
| Thrombocytes | 13 | 11 | 3 | 7 | 6 | 2 |
| | | | | *No* | | *Yes* |
| Granulocytopenic fever | | | | 32 | | 10 |
| Transiently reduced consciousness | | | | 37 | | 5 |
| Peripheral neuropathy | | | | 34 | | 8 |
| Treatment schedule modifications | | | | | | |
| Dose reduction | | | | 17 | | 25 |
| Delay of cycle | | | | 28 | | 14 |

[a]Number of patients.

**Table IV** Radiotherapy of previously untreated 'high-risk' patients with advanced seminoma[a]

| Reference | No. patients | Stage | Relapse | Survival (%) |
|---|---|---|---|---|
| Ball *et al.* (1982) | 14 | IID | NA[b] | 78 |
| Thomas *et al.* (1982) | 12 | IID | 5 | NA[b] |
| Schultz *et al.* (1984) | 17 | III + IV | 5 | 66 |
| Smalley *et al.* (1985) | 12 | IID | NA[b] | 100 |
| Mason and Kearsley (1988) | 12 | IID | 4 | 92 |
| Smalley *et al.* (1990) | 5 | IIID | 2 | NA[b] |
| Dosmann and Zagars (1993) | 13 | IID | 4 | 70 |

[a]Only series for which the present high-risk criteria were identifiable. [b]NA, not available.

**Table V** Treatment of 'high-risk' patients with advanced seminoma, cisplatin-based chemotherapy

| Reference[a] | No. patients | 'High-risk' characteristic | Chemotherapy[b] | Failure | Survival[c] (%) |
|---|---|---|---|---|---|
| Wettlaufer (1984) | 12 | III + IV | Cy OP | 1 | 94 |
| Friedman *et al.* (1985) | 6 | Previous treatment | PVB | 3 | 83 |
| Pizzocaro *et al.* (1986) | 12 | IID | PVB + A | | 75 |
| | 3 | III | BEP – A | | (2/3) |
| | 6 | IV | PVB, BEP | | (2/3) |
| Loehrer *et al.* (1987) | 33 | Radiotherapy | PVB, BEP | 10 | NA |
| Fosså *et al.* (1987) | 15 | Radiotherapy | PVB | 6 | 50* |
| Wilkinson *et al.* (1988) | 13 | III | BEP/Cy | | 69* |
| | 16 | IV | | | 33* |
| Clemm *et al.* (1989) | 7 | Radiotherapy | VIP | 2 | (5/7) |
| Horwich *et al.* (1992) | 22 | Radiotherapy | Carboplatin | | 95 |
| Schmoll *et al.* (1993) | 11 | IID | | 2 | NA |
| | 12 | III | Carboplatin | 3 | NA |
| | 3 | IV | | 2 | NA |
| Mencel *et al.* (1994) | 33 | Extragonadal | Cisplatin-based[d] | | 100 |
| | 18 | Radiotherapy | | | 72 |
| | 24 | IV | | | 79 |

[a]Only those series from the last 11 years for which the present 'high-risk' categories were identifiable are included. [b]Cy, cyclophosphamide; O, oncovin; P, platinum; V, vinblastine; B, bleomycin; E, etoposide; A, adriamycin; Carbo, carboplatin. [c]Cancer-specific survival, except * crude survival. [d]Various combinations.

when the present phase II study was initiated, no generally accepted high-risk criteria for seminoma existed and the eligibility criteria had to be arbitrarily defined with some support from the literature (Fosså *et al.*, 1987; Loehrer *et al.*, 1987). However, early reports on recurrent seminoma patients probably include previously irradiated patients with more advanced relapses and thus a high risk of failure of salvage treatment compared with those entered in the present trial.

The optimal combination chemotherapy in high-risk seminoma patients remains a matter of debate. Clinical experience with bleomycin in elderly patients has taught that this drug may be associated with a high-risk of pulmonary complications, in particular if prior mediastinal irradiation has been applied (Lehne and Lote, 1984). Wettlaufer (1984) has designed a combination drug schedule in which bleomycin is replaced by vincristine, a drug with minimal bone marrow toxicity. The present HOP regimen represents a modification of the Wettlaufer regimen, in which cyclophosphamide is replaced by ifosphamide, and which has shown high activity in germ cell cancer. Compared with the relapse and survival rates from Table IV and V, our 90% cancer-specific survival rate demonstrates a high efficacy of the chosen chemotherapy combination.

The study has, however, also indicated considerable toxicity of the HOP regimen: a 65-year-old man refused further treatment after the first cycle because of general deterioration (performance status WHO 4) and one patient died of toxicity. Grade 3 and 4 myelosuppression was observed at least once in 85% of the patients. The high incidence of severe haematopoetic side-effects has to be balanced against the Royal Marsden Hospital's (Horwich *et al.*, 1992) and Schmoll *et al.*'s (1993) experience with relatively non-toxic carboplatin monotherapy. These investigators obtained excellent final results with single-drug carboplatin chemotherapy in patients with metastatic seminoma. On the other hand, a 30% relapse rate was demonstrated. Such a high recurrence rate is felt unacceptable by many clinicians.

Several authors have expressed concern about chemotherapy tolerability after previous radiotherapy for seminoma (Pizzocaro *et al.*, 1986; Motzer *et al.*, 1988). However, according to Pizzocaro *et al.* (1986) and Fosså and Aamdal (1992), patients who have received infradiaphragmatic radiotherapy alone tolerate cisplatin-based chemotherapy as well as previously untreated patients. This is also our experience with the HOP chemotherapy. This observation is important as it does not contradict the present recommendation of infradiaphragmatic radiotherapy as standard treatment for seminoma stage I. Whether the combination of moderate-dose infradiaphragmatic radiotherapy and subsequent salvage chemotherapy in relapsing patients leads to an increased risk of long-term toxicity remains to be shown in future studies.

The role of routine consolidation treatment after chemotherapy for advanced seminoma has been intensively discussed (Friedman *et al.*, 1985; Fosså *et al.*, 1987; Motzer *et al.*, 1987; Ellison *et al.*, 1988; Wilkinson *et al.*, 1988; Horwich *et al.*, 1992). Some authors have recommended the routine removal of residual masses a few months after chemotherapy (Motzer *et al.*, 1987). Others (Schultz *et al.*, 1989; Horwich *et al.*, 1992) have advised observation or radiotherapy to increase the chance of local control. In series which consider post-chemotherapy surgery, 85–90% of the residual masses contain fibrosis and necrosis. As post-chemotherapy surgery in patients with advanced seminoma represents a surgical procedure with a relatively high risk of per- and post-operative complications, it does not seem justified to operate immediately in *all* patients in whom masses persist after chemotherapy. The present series supports this recommendation. Patients with lesions persisting after chemotherapy can as a rule be safely observed for up to 1 year, allowing further shrinkage of the mass. Though the present series comprises only ten evaluable patients, this recommendation also refers to residual tumours with a diameter ⩾30 mm after HOP chemotherapy. Lesions of this size have, in some authors' experience, been proven particularly often to contain residual malignancy (Motzer *et al.*, 1987). In our experience three of three resected residual retroperitoneal masses of that size and one mediastinal lesion contained complete necrosis. None of the six patients with long-term persisting retroperitoneal or mediastinal tumours without consolidation treatment have relapsed after a median observation time of 2.5 years.

Our results underline the difficulties in defining clinical criteria of response to chemotherapy in patients with advanced seminoma. Residual masses persist frequently and are often unsuitable for surgery. Furthermore, a clinical PR often represents a pathological CR. Our experience also indicates that clinicians do not believe in the significance of fine-needle biopsies, which may miss tiny tumour foci in a large fibrotic mass. If possible, a bidimensionally measurable response should not be assessed before 6 months has elapsed, allowing long-term post-chemotherapy shrinkage. The efficacy of chemotherapy in advanced seminoma should, however, preferably be evaluated by more meaningful biological parameters: time to progression and cancer-related survival.

In conclusion, the HOP chemotherapy regimen is highly effective in patients with bulky metastatic seminoma (stage ⩾IID, patients relapsing after radiotherapy, extragonadal presentation), but represents a relatively toxic treatment. Less toxic regimens are probably to be preferred in patients with low-volume disease. Previous infradiaphragmatic moderate-dose radiotherapy for seminoma does not imply a particularly high risk of complications. After HOP chemotherapy routine post-chemotherapy consolidation treatment (surgery, radiotherapy) seems unnecessary in the overwhelming majority of the patients. According to our ongoing observations shrinkage of residual masses can be expected for at least 1 year following chemotherapy.

**Acknowledgements**
The following members of the EORTC GU Group also participated in the above trial: A von Oosterom, University Hospital, Antwerp, Belgium; JJ Croles, William Alexander Hospital, GV den Bosch, The Netherlands; TAW Splinter, Erasmus University Rotterdam, The Netherlands; RFP van Velthoven, Institute Jules Bordet, Brussels, Belgium; CHN Veenhof, Academic Centre, Amsterdam, The Netherlands; PHM de Mulder, St. Radboud Hospital, Nejmegen, The Netherlands; F Keuppens, Academic Hospital, Free University, Brussels, Belgium; S Rodenhuis, The Netherlands Cancer Institute, Amsterdam, The Netherlands; C Vendrik, Academic Hospital, Utrecht, The Netherlands; C Theodore, Institute Gustave Roussy, Villejuif, France; JH Schornagel, Antoni van Leeuwenhoek Huis, Amsterdam, The Netherlands.

### References

BALL D, BARRETT A AND PECKHAM MJ. (1982). The management of metastatic seminoma testis. *Cancer*, **50**, 2289–2294.

BOSL GJ, GELLER NL, BAJORIN D, LEITNER SP, YAGODA A, GOLBEY RB, SCHER H, VOGELZANG NJ, AUMAN J, CAREY R, FAIR WR, HERR H, MORSE M, SOGANI P AND WHITMORE Jr W. (1988). A randomized trial of etoposide + cisplatin versus vinblastine + bleomycin + cisplatin + cyclophosphamide + dactinomycin in patients with good-prognosis germ cell tumors. *J. Clin. Oncol.*, **6**, 1231–1238.

CLEMM C, HARTENSTEIN R, WILLICH N, LEDDEROSE G AND WILMANNS W. (1989). Combination chemotherapy with vinblastine, ifosfamide and cisplatin in bulky seminoma. *Acta Oncol.*, **28**, 231–235.

DOSMANN MA AND ZAGARS GK. (1993). Postorchiectomy radiotherapy for stages I and II testicular seminoma. *Int. J. Radiat. Oncol. Biol. Phys.*, **26**, 381–390.

EINHORN LH, WILLIAMS SD, LOEHRER PJ, BIRCH R, DRASGA R, OMURA G AND GRECO FA. (1989). Evaluation of optimal duration of chemotherapy in favorable-prognosis disseminated germ cell tumors: a Southeastern Cancer Study Group protocol. *J. Clin. Oncol.*, **7**, 387-391.

ELLISON MF, MOSTOFI FK AND FLANIGAN RC. (1988). Treatment of the residual retroperitoneal mass after chemotherapy for advanced seminoma. *J. Urol.*, **140**, 618-620.

FOSSÅ SD AND AAMDAL S. (1992). Toxicity of combined chemotherapy and radiotherapy: clinical aspects. In *Combined Radiotherapy and Chemotherapy in Clinical Oncology*, Horwich A (ed.) pp. 34-39. Edward Arnold: London.

FOSSÅ SD, BORGE L, AASS N, JOHANNESSEN NB, STENWIG AE AND KAALHUS O. (1987). The treatment of advanced metastatic seminoma: experience in 55 cases. *J. Clin. Oncol.*, **5**, 1071-1077.

FRIEDMAN EL, GARNICK MB, STOMPER PC, MAUCH PM, HARRINGTON DP AND RICHIE JP. (1985). Therapeutic guidelines and results in advanced seminoma. *J. Clin. Oncol.*, **3**, 1325-1332.

HORWICH A, DEARNALEY DP, A'HERN R, MASON M, THOMAS G, JAY G AND NICHOLLS J. (1992). The activity of a single-agent carboplatin in advanced seminoma. *Eur. J. Cancer*, **28A**, 1307-1310.

LEHNE G AND LOTE K. (1984). Pulmonary toxicity of cytotoxic and immunosuppressive agents. A review. *Acta Oncol.*, **29**, 113-123.

LOEHRER PJ, BIRCH R, WILLIAMS SD, GRECO FA AND EINHORN LH. (1987). Chemotherapy of metastatic seminoma: the Southeastern Cancer Study Group experience. *J. Clin. Oncol.*, **5**, 1212-1220.

MACKENZIE AR. (1966). Chemotherapy of metastatic seminoma. *J. Urol.*, **96**, 790-793.

MASON BR AND KEARSLEY JH. (1988). Radiotherapy for stage 2 testicular seminoma: the prognostic influence of tumor bulk. *J. Clin. Oncol.*, **6**, 1856-1862.

MEDICAL RESEARCH COUNCIL WORKING PARTY ON TESTICULAR TUMOURS. (1985). Prognostic factors in advanced non-seminomatous germ-cell testicular tumours: results of a multicentre study. *Lancet* **i**, January **5**, 8-11.

MENCEL P, MOTZER RJ, MAZUMDAR M, VLAMIS V, BAJORIN DF AND BOSL GJ. (1994). Advanced seminoma: treatment results, survival, and prognostic factors in 142 patients. *J. Clin. Oncol.*, **12**, 120-126.

MOTZER RJ. (1993). Optimal treatment for advanced seminoma? *Cancer*, **72**, 3-4.

MOTZER R, BOSL G, HEELAN R, FAIR W, WHITMORE W, SOGANI P, HERR H AND MORSE M. (1987). Residual mass: an indication for further therapy in patients with advanced seminoma following systemic chemotherapy. *J. Clin. Oncol.*, **5**, 1064-1070.

MOTZER RJ, BOSL GJ, GELLER NL, PENENBERG D, YAGODA A, GOLBEY R, WHITMORE WF, FAIR WR, SOGANI P, HERR H, MORSE M, CAREY RW AND VOGELZANG N. (1988). Advanced seminoma: the role of chemotherapy and adjunctive surgery. *Ann. Intern. Med.*, **108**, 513-518.

PECKHAM MJ, BARRETT A, MCELWAIN TJ AND HENDRY WP. (1979). Combined management of the malignant teratoma of the testis. *Lancet*, **ii**, 267-270.

PIZZOCARO G, SALVIONI R, PIVA L, ZANONI F, MILANI A AND FAUSTINI M. (1986). Cisplatin combination chemotherapy in advanced seminoma. *Cancer*, **58**, 1625-1629.

SCHMOLL H-J, HARSTRICK A, BOKEMEYER C, DIECKMANN K-P, CLEMM C, BERDEL WE, SOUCHON R, SCHÖBER C, WILKE H AND POLIWODA H. (1993). Single-agent carboplatinum for advanced seminoma. *Cancer*, **72**, 237-243.

SCHULTZ HP, VON DER MAASE H, RØRTH M, PEDERSEN M, SANDBERG NILSEN E, WALBOM-JØRGENSEN S AND DATECA STUDY GROUP. (1984). Testicular seminoma in Denmark 1976-1980. Results of treatment. *Acta Radiol. Oncol.*, **23**, 263-270.

SCHULTZ SM, EINHORN LH, CONCES DJ, WILLIAMS SD AND LOEHRER PJ. (1989). Management of postchemotherapy residual mass in patients with advanced seminoma: Indiana University experience. *J. Clin. Oncol.*, **7**, 1497-1503.

SMALLEY SR, EVANS RG, RICHARDSON RL, FARROW GM AND EARLE JD. (1985). Radiotherapy as initial treatment for bulky stage II testicular seminomas. *J. Clin. Oncol.*, **3**, 1333-1338.

SMALLEY SR, EARLE JD, EVANSE RG AND RICHARDSON RL. (1990). Modern radiotherapy results with bulky stages II and III seminoma. *J. Urol.*, **144**, 685-689.

STOTER G, SYLVESTER R, SLEIJFER DT, TEN BOKKEL HUININK ?, KAYE SB, JONES WG, VAN OOSTEROM AT, VENDRIK CPJ, SPAANDER P AND DE PAUW M. (1987). Multivariate analysis of prognostic factors in patients with disseminated nonseminomatous testicular cancer: results from a European Organization for Research on Treatment of Cancer multiinstitutional phase III study. *Cancer Res.*, **47**, 2714-2718.

THOMAS G. (1991). Management of metastatic seminoma: role of radiotherapy. In *Testicular Cancer – Clinical Investigation and Management*, Horwich A (ed.) pp. 211-231. London: Chapman & Hall Medical.

THOMAS GM, RIDER WD, DEMBO AJ, CUMMINGS BJ, GOSPODAROWICZ M, HAWKINS NV, HERMAN JG AND KEEN CW. (1982). Seminoma of the testis: results of treatment and patterns of failure after radiation therapy. *Int. J. Radiat. Oncol. Biol. Phys.*, **8**, 163-174.

WETTLAUFER JN. (1984). The management of advanced seminoma. *Semin. Urol.*, **2**, 257-263.

WILKINSON PM, READ G AND MAGEE B. (1988). The treatment of advanced seminoma with chemotherapy and radiotherapy. *Br. J. Cancer*, **57**, 100-104.